# Atrioventricular Dissociation With Ischemic-Appearing ST-Segment Changes

**DOI:** 10.1016/j.jaccas.2026.108039

**Published:** 2026-05-20

**Authors:** Ziad Hindosh, Elliot Roufeh, Justin Hayase, Kaylin Nguyen

**Affiliations:** a Olive View-UCLA Medical Center, Department of Medicine, Division of Cardiology; b UCLA Cardiac Arrhythmia Center, David Geffen School of Medicine at UCLA

**Keywords:** acute heart failure, cardiomyopathy, electrocardiogram, heart block, myocarditis

## Abstract

**Background:**

ST-segment elevation in a wide-complex rhythm may signal acute coronary occlusion, a critical cannot-miss diagnosis, but also brings a broad differential with diagnostic and triaging challenges, especially in patients presenting with shock.

**Case Summary:**

A 46-year-old woman with unknown past medical history presented with fever, chest pain, and hemodynamic instability concerning for shock. Electrocardiogram demonstrated high-grade atrioventricular block with a wide-complex ventricular rhythm and anterior ST-segment changes, raising concern for acute coronary syndrome. Emergent coronary angiography revealed normal coronary arteries, and she was found to have severe left ventricular systolic dysfunction consistent with fulminant myocarditis complicated by diffuse conduction disease with an accelerated idioventricular rhythm.

**Discussion:**

Diffuse myocardial inflammation from fulminant myocarditis can disrupt atrioventricular conduction and cause ischemic-appearing ST-segment changes, signifying a poor prognosis.

**Take-Home Messages:**

There is a broad differential for ST-segment abnormalities, and fulminant myocarditis should be considered in patients with inflammatory symptoms, conduction disease, and cardiogenic shock after exclusion of acute coronary syndrome.

## Graphical Abstract

**Figure:**
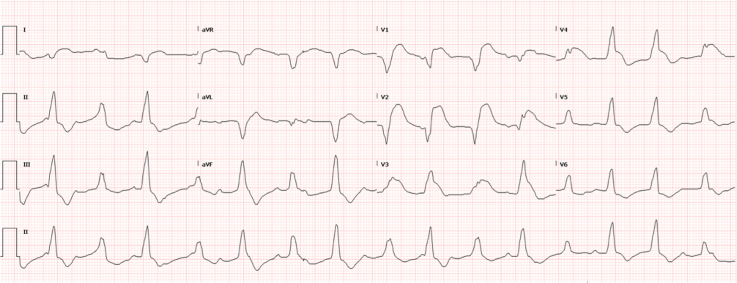

## Case

A 46-year-old woman with unknown past medical history presented to a county hospital with 1 day of intermittent left-sided chest pressure that radiated to her back, preceded by 4 days of fevers, chills, nausea, and emesis. On arrival, vital signs were a temperature of 38.2 °C, heart rate of 102 beats per minute, blood pressure of 82/47 mm Hg, and pulse oximetry of 95%. Troponin-I was elevated to 80.923 ng/mL. An electrocardiogram was obtained (Figure 1, Video 1).

**Take-Home Messages**

• Recognize atrioventricular dissociation and ventricular escape rhythms in diffuse conduction disease and understand how myocardial inflammation or injury can produce ischemic-appearing changes in wide-complex rhythms.
• Identify high-risk electrocardiographic features in conjunction with clinical presentation to guide differential diagnosis, management, and critical triage decisions.

**Figure 1:**
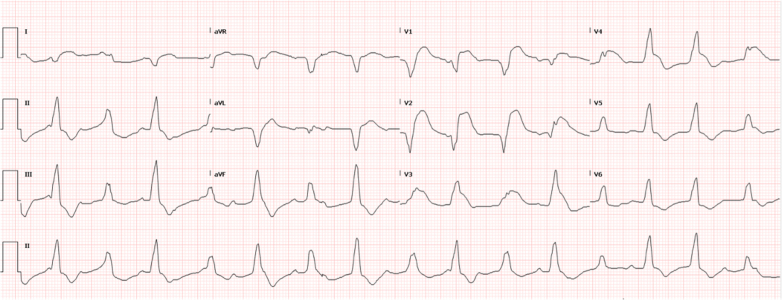
12-Lead Electrocardiogram Obtained Upon Arrival Demonstrating Atrioventricular Dissociation With a Wide-Complex Ventricular Rhythm and ST-Segment Elevation P waves are present at a constant interval but do not have a fixed relationship with the QRS complexes, indicating a high-grade atrioventricular block. There is a discordant ST-segment elevation of >5 mm in the anteroseptal leads (V_1_-V_2_) and concordant ST-segmentation elevation in the anterior leads (v_3_-v_4_), consistent with Sgarbossa's criteria, creating an ischemic-appearing pattern. This electrocardiogram illustrates a high-risk presentation that requires diagnostic evaluation.

What is the rhythm of the electrocardiogram?

A. Atrial fibrillation with left bundle branch block
B. Atrial flutter with 2:1 conduction
C. Sinus tachycardia with a Mobitz type I atrioventricular block with a left bundle branch block
D. Sinus tachycardia with high-grade atrioventricular block and accelerated idioventricular rhythm
E. Ventricular tachycardia

## Discussion

This electrocardiogram demonstrates high-grade atrioventricular block (AVB) with atrioventricular dissociation, characterized by an atrial rate of approximately 150 beats/min (beats/min) and an accelerated idioventricular rhythm (AIVR) at approximately 90 bpm. AIVR is an ectopic ventricular rhythm that occurs at faster-than-expected ventricular escape rates of 30 to 40 bpm but slower than ventricular tachycardia.^1^ While atrioventricular dissociation is a known feature of AIVR, it is not always visualized on a 12-lead electrogram. The presence of 2 distinct QRS morphologies suggests multiple ventricular foci, supporting diffuse myocardial involvement.

Wide QRS rhythms such as AIVR are often associated with secondary repolarization abnormalities, resulting in ST-segment deviations opposite the direction of the QRS complex. However, this tracing demonstrates discordant ST-segment elevation >5 mm in lead V_2_, a finding associated with acute coronary occlusion in the setting of a left bundle branch block and incorporated into the Sgarbossa criteria.^2^ Although Sgarbossa criteria have not been validated in AIVR, discordant ST-segment elevation—particularly when accompanied by chest pain, hemodynamic instability, and elevated cardiac biomarkers—remains physiologically concerning and appropriately prompted emergent coronary angiography. This patient was transferred to a ST-segment elevation myocardial infarction receiving center for a left-heart catheterization, which revealed normal coronary arteries. She was found to have severe left ventricular systolic dysfunction with an ejection fraction of 10% and required multiple inotropes and vasopressors. She was subsequently transferred to an advanced heart failure center for evaluation of advanced therapies, including heart transplantation.

The differential diagnosis included ST-segment elevation myocardial infarction, Takotsubo cardiomyopathy, and infiltrative or inflammatory cardiomyopathies such as sarcoidosis or Lyme carditis. Ultimately, the patient was diagnosed with probable fulminant myocarditis based on elevated cardiac biomarkers, diffuse conduction system involvement, exclusion of obstructive coronary artery disease, and a preceding viral prodrome.^3^

This case highlights the importance of recognizing high-risk electrocardiographic and clinical features in patients presenting with ST-segment elevation and shock. Early recognition of this electrocardiographic pattern should prompt emergent coronary angiography to exclude acute coronary occlusion and, once ischemia is ruled out, immediate consideration of alternative diagnoses such as myocarditis. Electrical instability—including high-grade AVB—together with chest pain, heart failure symptoms, and hemodynamic instability represent a constellation of findings classically associated with fulminant myocarditis. High-risk features such as high-grade AVB, QRS duration >120 ms, left bundle branch block, and malignant ventricular arrhythmias are associated with severe left ventricular dysfunction and poor clinical prognosis.^3^ Recognition of these features should prompt early triage to an advanced heart failure center where specialized myocarditis expertise and mechanical circulatory support are available.

## Funding Support and Author Disclosures

The authors have reported that they have no relationships relevant to the contents of this paper to disclose.

## Appendix

Video 1 Electrocardiogram analysis demonstrating atrioventricular dissociation and ischemic appearance.
